# Announcement: 33rd International Conference on Coordination Chemistry

**DOI:** 10.1155/MBD.1997.249

**Published:** 1997


					33RD INTERNATIONAL
CONFERENCE ON
COORDINATION CHEMISTRY
The Chemistry of Metal Ions in Everyday Life
30th August to 4th September 1998, Florence, Italy
http://risc3. rm.fi.cnr.it:8002/
Plenary lecturers
Claudio Bianchini, Firenze, Italy
"Liquid Biphase Catalysis with Polyphosphine Metal Complexes"
Kim R. Dunbar, Michigan, USA
Odile Eisenstein, Montpellier, France
Harry B. Gray, Pasadena, CA, USA
"Forty Years of Metal, Ligands, Electrons and Proteins
Hiizu Iwamura, Fukuoka, Japan
"Heterospin Magnets Made of Transition Metals Ions with Poly(aminoxyl) Radicals as Bridging
Ligands"
Steve Lippard, MIT, MA, USA
Robin N. Perutz, York, UK
"Photochemistry of Metal Dihydride Complexes"
Lawrence Que, Minnesota State Univerity, USA
Alex von Zelewsky, Fribourg, Switzerland
"Stereoselective Synthesis of Coordination Compounds"
The View of a Nobel Laureate
Jean-Marie Lehn, University of Strasbourg, France
Special lecturers: "The way we were"
Fred Basolo, Northwesten University, IL, USA
Stanley Kirschner, Wayne University, MI, USA
"ICCC Adventures: Going Way Back"
Luigi Maria Venanzi, ETH, Zurich, Switzerland
Shoichiro Yamada, Osaka University, Japan
249
Minisymposia and session lectures: Tentative list of speakers
Supramolecular Chemistry
Didier Astruc, Bordeaux, France
Vincenzo Balzani, Bologna, Italy
Paul D. Beer, Oxford, UK
Peter Belser, Fribourg, Switzerland
Carlo Alberto Bignozzi, Ferrara, Italia
Sebastiano Campagna, Messina, Italy
Edwin C. Constable, Basel, Switzerland
Luisa De Cola, Bologna, Italy
A. P. de Silva, Belfast, North Ireland, UK
Gianfranco Denti, Pisa, Italy
Luigi Fabbrizzi, Pavia, Italy
"Supramolecular Switches and Machines
Donald Fitzmaurice, Dublin, Ireland
Lucia Flamigni, Bologna, Italy
Michael Graetzel, Lausanne, Switzerland
Masa-aki Haga, Kamihama, Japan
Olivier Kahn,,Pessac, France
Jean-Pierre Launay, Toulouse, France
Jean-Pierre Majoral, Toulouse, France
Thomas E. Mallouk, Penn State, PA, USA
John McCleverty, :Bristol, UK
"Chemionics: Molecular Electronics, Molecular Ionics, Non-linear Optics
Thomas J. Meyer, Chapel Hill, NC, USA
Daniel G. Nocera, Michigan State, MI, USA
"Optical Supramolecules for Chemical and Physical Sensing
Jean Pierre Sauvage, Strasbourg, France
Franco Scandola, ~Ferrara, Italy
Fraser J. Stoddart, Birmingam, UK
Michael J. Therien, Philadelphia, PA, USA
Nicholas J. Turro, New York, NY, USA
Margherita Venturi, Bologna, Italy
Fritz Vogtle, Bonn, Germany
"New Functional Dendrimers
Jeffrey Zink, Los Anngeles, CA, USA
Bioinorganic Chemistry
Shigetoshi Aono, Ishikawa, Japan
"Structure and function of carbon monoxide dependent transcriptional regulator, CooA
Lucia Banci, Firenze, Italy
Gerard W. Canters, Leiden, The Netherlands
David Case, La Jolla, CA, USA
Luigi Casella, Pavia, Italy
"Structure-activity Relationship in Polynuclear Copper Enzymes
Tim Clark, Erlangen, Germany
Dimitri Coucovannis, Ann Arbor, MI, USA
James A. Cowan, Harward, MA, USA
"Protein-Bound Iron-Sulfur Clusters. Reactivity and Mechanism of Assembly"
Sture Forsen, Lund, Sweden
Allen H. Hill, Oxford, UK
"The Investigation of Proteins by Scanning Probe Microscopy"
Lian-Nian Ji, Guangzhou, China
"Structures and Function of Two Types of Copper in Nitrite Reductase"
Kenneth D. Karlin, Baltimora, MD, USA
"Copper-Dioxygen Interactions and Models for Heme-Copper Oxidases
Peter Kollmann, San Francisco, CA, USA
250
Lechoslaw Latos-Grazynski, Wroclav, Poland
"Novel routes of the iron porphyrin modifications
Anjos Macedo, Oeiras, Portugal
Bo Malmstrom, Goteborg, Sweden
"Effects of Protein Folding on Metalloprotein Redox-active Sites
Bernard Meunier, Toulouse, France
"Recent advances in oxidation reactions mediated by biomimetic catalysts"
Vincent L. Pecoraro, Ann Arbor, MI, USA
Alexander Ryabov, Moscow, Russia
Per Siegbahn, Stockholm, Sweden
Edward I. Solomon, Stanford, CA, USA
Shinnichiro Suzuki, Osaka, Japan
"Structures and Function of Two Types of Copper in Nitrite Reductase
William B. Tolman, Minneapolis, MN, USA
Heinrich Vahrenkamp, Freiburg, Germany
"Alcohols and Aldehydes in the Ligand Sphere of the Zinc Attempts ad Modelling Alcohol
Dehydrogenase
Alejandro Vila, Rosario, Argentina
Anna Walker, Tucson, AZ, USA
Karl Wieghardt, Muhleim, Germany
"Coordination Chemistry of Phenoxyl and Thyil Radicals
Jay R. Winkler, Pasadena, CA, USA
"Electron Transfer in Metalloproteins: Distance, Dynamics and Folding
Transition Metal Ions in Material Chemistry
Lidia Armelao, Padova, Italy
Charles Bauschlicher, NASA, CA, USA
M. L. Boilot, Orsay, France
Enric Canadell, Barcellona, Spain
Patrick Cassoux, Tolouse, France
George Christou, Bloomington, IN, USA
Peter Day, London, UK
Philippe Gutlich, Mainz, Germany
Jaap G. Haasnoot, Leiden, The Netherlands
"The Synthesis of Spincrossover Complexes of Iron(ll) with 1,2,4-triazoles. X-ray Structures
A. Hauser, Geneve, Switzerland
James R. Ibers, Evanston, IL, USA
V. Leonard Interrante, Troy, NY, USA
Mercouri Kanazidis, Michigan, MI, USA
Hayao Kobayashi, Okazaki, Japan
Jing Li, Camden, NJ, USA
"Low Dimensional Inorganic Solids: Crystal Growth and Characterization of Novel Metal
Chalcogenides
W. Linert, Wien, Austria
Achim Muller, Bielefeld, Germany
Jean Rouxel, Nantes, France
Renato Ugo, Milano, Italy
"Non Linear Optical Properties of Organometallic Complexes and Porphyrins
Michael Verdaguer, Paris, France
Jack Williams, Argonne, IL, USA
Yuri, V. Yablakov, Kazan, Russia
Masahiro Yamashita, Nagoya, Japan
"Quasi-One-Dimensional Halogen-Bridged Ni(lll) Complexes and Haldane Gap Systems"
251
Organometallic Chemistry
Rumiya Abdereimova, Alma-ata, Kazakhstan
Thomas Albright, Houston, TX, USA
"The Mechanism of the Butadiene Dimerization Reaction
Alan Balch, Davis, CA, USA
John E. Bercaw, Caltech, LA, CA, USA
"Some Aspects of the Organometallic Chemistry Underlying Natta-Ziegler Polymerization of
Olefins"
Maria Jose Calhorda, Lisboa, Portugal
Ernesto Carmona, Sevilla, Spain
"Reactivity Studies on Cationic Alkylidene Complexes of Rhodium and Iridium
Ken G. Caulton, Bloomington, IN, USA
"New Insights and Applications of Transition Metal Hydrides"
Jwu-Ting Chen, Taipei, Taiwan
"Allenyl/Propargy Metal Complexes Mediated Versatile Bond-Formation
Peter Comba, Heidelberg, Germany
Dainis Dakternieks, Geelong, Australia
"Organotin Chemistry: Strategies to Chirotechnology and New Catalysts
Pierre H. Dixneuf, Rennes, France
"Ruthenium Catalysts and C-C Bond Formation
Kees Elsevier, Amsterdam, The Netherlands
Jose Gimeno, Oviedo, Spain
"Activation of Terminal Alkynes: Efficient Approaches to the Selective Syntesis of Highly
Unsaturated Hydrocarbon Chains"
John Gladysz, Salt Lake City, Utah, USA
"New Catalytic and Stoichiometric Carbon-Carbon Bond Forming Reactions in Metal Coordination
Spheres
Lai Yoong Goh, Kuala Lumpur, Malaysia
"Group 15 and Group 16 Polyatomic Molecules in Organochromium Chemistry"
Jurgen Heck, Hamburg, Gerrmany
Guochen Jia, Hong Kong, Hong Kong
"Synthesis and Characterization of some Dihydrogen/hydride Complexes of Ruthenium and
Osmium, and Their Roles in Catalytic Hydrogenation Reactions"
Brian F. G. Johnson, Cambridge, UK
Klaus Jurkshat, Dortmund, Germany
"lntramolecular Coordination in the Chemistry of IV Elements"
Alfred B. P. Lever, Toronto, Canada
David Milstein, Rehovot, Israel
"Metal Insertion into carbon-carbon single bonds"
Ilya I. Moiseev, Moscow, Russia
"Reactions of Low-Valence Palladium Clusters"
Thomas Rauchfuss, Urbana, IL, USA
"Organometallic Models for Hydrogenating Catalysis"
Otto J. Scherer, Kaiserslautern, Germany
"'Naked' Pn and Sn Ligands: A Novel Chapter in Coordination Chemistry"
Richard R. Schrock, Cambridge MIT, MA, USA
"New Group 4 Catalysts for the Living Polymerization of Terminal Olefins
John Shapley, Urbana, IL, USA
"Metal Cluster Complexes of Fullerenes
Edward I. Stiefel, Exxon, Annandale, NJ, USA
"Internal Electron Transfer in Transition Metal Chalcogenide Systems
Kohei Tamao, Kyoto, Japan
"Pentacoordinate Fluorosiliconate Oligomers
Kazuyo Tatsumi, Nagoya, Japan
"A New Entry into the Group 6 Transition Metal Chalcogenide Chemistry"
Hubert Wadepohl, Heidelberg, Germany
Joseph Ziolkowsky, Wroclaw, Poland
"Perspectives of Rhodium Organometallic Catalysis. Fundamental and Applied Aspects
252
Solution Chemistry
Silvio Aime, Torino, Italy
Pradyat Banerjee, Calcutta, India
"lnteration of Nitrogen Bases with Some Platinum(ll) and Palladium(ll) Complexes Usual and
Unusual
Sue Berner-Price, Nathan, Australia
"Solution NMR Studies of Metal-Based Drugs
Cynthia J. Burrows, Salt Lake City, Utah, USA
"Interaction of Nickel Peptides with DNA: Recognition, Clevage and Cross-linking
Daryle H. Busch, Kansas, USA
Steve Dunham, MIT, Cambridge, MA, USA
Timothy J. Egan, Cape Town, South Africa
Thomas Kaden, Basel, Switzerland
Eiichi Kimura, Hiroshima, Japan
"Interaction of Macrocyclic Complexes with Nucleic Acids to Control Gene Expression
Bernhardt Lippert, Dortmund, Germany
Claudio Luchinat, Firenze, Italy
Kazuko Matsumoto, Tokyo, Japan
"Pursuit of Platinum Complexes That Interact with Nucleic Acid in a New Mode"
Thomas J. Meade, Pasadena, CA, USA
Dan Meyerstein, Beer-Sheeva, Israel
K. N. Raymond, Berkeley, USA
Jan Reedijk, Leiden, The Netherlands
"Supramolecular Aspects of Metal-containing Drugs
Peter J. Sadler, London, UK
Roberto Sanchez-Delgado, Caracas, Venezuela
"Metal Complexes with Nitrogen Ligands as Chemotherapeutic Agents Against Tropical Diseases
Dieter Sellmann, Erlangen, Germany
"Chemical Principles, Structural Blue-prints, and the Relevance of Iron Sulfur Complexes for N2
Fixation
Helmut Sigel, Basel, Switzerland
Einar Sletten, Bergen, Norway
"Stabilisation of Triple-Stranded DNA Oligomers by Transition Metal Ions
Wenxia Tang, Nanjing, China
"dentification and Structural Characterization of Some Platino and Pallado-Metallothionein
Fulvio Uggeri, Bracco spa, Milan, Italy
Kui Wang, Beijing, China
"The chirality effect of the metal complexes in their transportation and bioactivity"
Lon J. Wilson, Houston, TX, USA
"Metallofullerene Drug Design"
Osamu Yamauchi, Nagoya, Japan
Metals in Life and Environment
Michele Aresta, Bari, Italy
Barbara K. Burgess, Irvine, CA, USA
Stefano Ciurli, Bologna, Italy
"Structure-function relationships in urease: the crystal structures of native and inhibited enzyme
Cristopher C. Cummins, MIT, Cambridge, MA, USA
William De W. Horrocks, Penn.State Univ., USA
"Yb3+ Chelates as Near Infrared Luminophores for Chemiosensor and Imaging Applications
Marcel Fontcave, Grenoble, France
Michael D. Fryzuk, Vancouver, Canada
"The Continuing Saga of Dinitrogen Activation
Masanobu Hidai, Tokyo, Japan
"Dinitrogen Activation and Its Chemical Transformation into Heterocyclic Compounds
Peter May, Murdoch, Australia
Douglas Rees, Caltech, Pasadena, CA, USA
253
Barry Smith, Norwich, UK
"Recent studies on the structure and function of Nitrogenase
A. G. Sykes, Newcastle, UK
"Reactivity of the Cull/Free-Radical Containing Enzyme Galactose Oxidase"
Rudi van Eldik, Erlangen, Germany
"Activation of Small Molecules by Transition Metal Centers in Biological and Environmental
Systems. A Mechanistic Approach
John Webb, Perth, Australia
"Biominerals, Medicine & Materials Science"
James Wild, Austin, TX, USA
David R. Williams, Cardiff, UK
For more information:
http://risc3. rm.fi.cnr.it:8002/
Department of Chemistry, University of Florence
Via Gino Capponi 7, 50121 Florence, Italy
Chairman: Professor Ivano Bertini
Phone: +39 55 2757549, Fax: +39 55 2757555
Secretary: Dr Maurizio Peruzzini
Phone: +39 55 245990, Fax: +39 55 2478366, email: ICCC@riscl.lrm.fi.cnr.it
254

				

